# A Bourdieusian Latent Class Analysis of Cultural, Arts, Heritage and Sports Activities in the UK Representative *Understanding Society* Dataset

**DOI:** 10.1177/00380385221130163

**Published:** 2022-11-13

**Authors:** Emma S Walker, Daisy Fancourt, Feifei Bu, Anne McMunn

**Affiliations:** University College London, UK; University College London, UK; University College London, UK; University College London, UK

**Keywords:** Bourdieu, capital, cultural engagement, latent class analysis, Omnivore hypothesis

## Abstract

To Bourdieu, interaction with culture has symbolic power and drives the manifestation of social stratification. Many have adapted his theory and methodology, developing new models of cultural engagement. Here, to further integrate these theoretical and methodological approaches, Bourdieu’s tools were used to operationalise and interpret a Latent Class Analysis of cultural engagement in the *Understanding Society* dataset. Six classes of increasing engagement were established, and were increasingly correlated with youth, capital and social advantage. However, some qualitative differences in engagement were also seen. The classes also varied by which characteristics correlated with membership. For example, economic capital was associated with sports engagement, while advantaged social position was associated with broad-scale engagement. Overall, this analysis combined Bourdieusian theory with contemporary methodology in the largest representative UK dataset and highlights the broader relevance of cultural engagement patterns in indicating (and possibly generating) status, identity, capital and social position.

## Introduction

Pierre Bourdieu’s impact on sociology cannot be understated (Bennett et al., 2008). Through interconnected theoretical tools the French sociologist set out insightful models that are outstanding in their applicability across a breadth of sociological enquiries (Bourdieu, 1981, 1986, 1990). Many contemporary analyses have used Bourdieu to explore cultural engagement. This broad, yet subjective term describes the production or consumption of arts and culture. This may include visual, musical, performance, literary, heritage or sporting activities that often represent the ideas, traditions or experiences of groups of people (Cambridge English Dictionary, 2020; Kroeber and Kluckhohn, 1952). In addition, cultural engagement might be used by individuals to indicate their social or cultural identities, or status (Bourdieu, 1981). Therefore, volume (and type) of cultural engagement is often shown to be associated with social advantage or position. Within sociology, Bourdieusian-influenced analyses conceptualise cultural engagement as a part of (or an indicator for) wider cultural and social resources (Bennett et al., 2008; Hanquinet et al., 2014; Julien, 2015; Roose, 2015). These works typically use Multiple Correspondence Analysis (MCA) to describe the spatial distance between social position and cultural preferences. Tastes are seen as reflexive indicators of, or mechanisms for, attaining social status. Grounded in Bourdieu’s capital theory, this work sees accumulated cultural resources as socially distinctive mechanisms for establishing distance between the members of a hierarchical society (Bourdieu, 1981; Carlisle et al., 2008; Costa and Murphy, 2015).

By contrast, American communitarian (Coleman, 1990; Granovetter, 1973) inspired research on cultural capital focuses on engagement (over tastes), conceptualises cultural resources as a public good (Julien, 2015) and aims to identify barriers to engagement. Publications typically use either regression (Devine and Dowds, 2013; Veenstra, 2007b) or Latent Class Analysis (LCA) (Bunting et al., 2008; Mak et al., 2020; Reeves, 2015) to demonstrate how factors such as education, gender or race (Bunting et al., 2008; Mak et al., 2020) may enable or prevent engagement. However, this literature often does not recognise engagement as a display, or distinction behaviour, and so does not consider the bidirectional and divisive influence of accumulated resources on engagement. Additionally, some of the publications are either collaborations with funding bodies (Arts Council England, 2016; Bunting et al., 2008) or use data that was designed to support further funding (Devine and Dowds, 2013; Mak et al., 2020; Reeves, 2015), which may introduce a significant bias.

Another key body of literature has made use of latent class methodology to develop Peterson’s Omnivore hypothesis (Peterson and Kern, 1996). Influenced by Bourdieu, the Omnivore hypothesis proposes that the most advantaged in contemporary societies consume broadly across all ‘brows’ of culture, enjoying for example, reality television alongside classical music. In an LCA, the underlying structure of a non-directly observable concept is established as a new, latent, categorical variable (McCutcheon, 1987). Therefore, these analyses have been able to define a specific omnivore class who are characterised by their broad cultural engagement. Investigators have then explored the economic and socio-demographic characteristics of this omnivorous group, identifying factors that determine membership. However, these LCAs have either focused on particular cultural fields, such as music (Chan and Goldthorpe, 2007; Leguina et al., 2016; Tampubolon, 2008), look exclusively at institutional attendance (Alderson et al., 2007; Chan and Goldthorpe, 2005; Katz-Gerro, 2002; Reeves, 2015; Sintas and Álvarez, 2002) or are models of for example, online participation (Van Steen et al., 2015) and interactions (Julien, 2015), culinary preferences (Kamphuis et al., 2015) or offer an updated conceptualisation of social class (Savage et al., 2013) using unrepresentative data (Mills, 2014).

Therefore, while Bourdieusian theory can be used to conceptualise diverse cultural engagement patterns as key status indicators, empirical investigations of this theoretical framing have not been conducted in a large, representative population sample in the UK. Thus, our understanding of how cultural activities may cluster together, or relate to different indicators of capital and social position is limited. Therefore, this study combines contemporary latent class methodology with Bourdieusian theory, while extending the lens of cultural engagement to generate a concise and unifying model of arts, culture, sports and heritage activities. This was achieved using a three-step, data-driven approach, where items were first scaled empirically so researcher bias within this highly subjective research area could be minimised. Second, the LCA model was established, and cultural engagement patterns described. The third section presents associations between engagement classes and social and economic capital, aspects of social position and identity.

Overall, the study offers a novel, theoretically informed, empirical description of social stratification and societal structure in the UK and provides sociologists with new insight into the social meaning of culture in contemporary society.

## Theoretical Background

### Capital, Field, Habitus and Symbolic Violence: The Role of Culture in Structuring Society

Established through the work of political economists such as Marx and Smith (Marx, 1867; Smith, 1776), capital theory describes how individuals and groups may accumulate, exchange or gain profit from economic resources. Bourdieu argued that the complexity of capital and power cannot be understood if only economic resources are considered and applied the capital framework to social resources, or symbolic, practical or emotional assets made available through social group membership. He further proposed that cultural objects, institutions and dispositions are forms of cultural capital that may be accumulated through a synergy between inherited attributes, socialisation and labour (Bourdieu, 1986). Thus, social agents have an accumulation of capital that determines their social position and enables them to build further capital (Bourdieu, 1986; Costa and Murphy, 2015).

However, unlike communitarian capital theorists, who frame capital as a cohesive social good that benefits all members of society (Coleman, 1990), a crucial feature of Bourdieu’s capital is its uneven distribution and subsequent ability to set people apart, benefiting only a distinct few (Bourdieu, 1981). Here, Bourdieu used his concepts of field, habitus and symbolic violence to describe how the concentration of capital in the hands of society’s elite enables them to establish and maintain their hegemonic position. A field is any social hierarchical structure upon which individuals compete for capital and subsequent position (Costa and Murphy, 2015; Mohr, 2013). A social agent’s ability to successfully compete results from the degree of complementarity between the field and their way of being, or their habitus. The habitus is therefore an ongoing social reflection that incorporates features of an individual’s social identity such as their age or gender, and their current and historical social context to determine their attitudes, approaches and practices (Costa et al., 2019). Atop of this conceptual space is Bourdieu’s schema of symbolic violence: a system of subtle, yet powerful oppression. Here, social agents communicate their capital to affirm their position while establishing psychosocial or practical barriers that prevent lower status groups from accessing capital, or from enhancing their social position (Carlisle et al., 2008). Symbols might include indicators of wealth, membership of exclusive social groups or accreditations from educational or occupational institutions. Similarly, cultural objects such as books (Wang et al., 2006), artwork (Silva, 2006) or sporting equipment (Abel, 2008; Veenstra, 2007b) and cultural competencies, skills or knowledge act to distinguish a person from their peers who do not have these forms of capital; drawing lines of distinction between positions on the field.

Key to Bourdieu’s work, as presented in his 1981 book, *Distinction*, are theories explaining how cultural tastes and practices become a characterising feature of socio-economically defined status groups (Bourdieu, 1981). For Bourdieu, it is specifically the uneven distribution of *cultural* capital that determines and drives societal stratification. Cultural capital is first established in the family setting, then cultivated through social interactions and educational systems. Therefore, it is a transgenerational mechanism, much like economic inheritance, through which social stratification is maintained and entrenched. This symbolically violent system of ‘cultural aristocracy’ (Bourdieu, 1980) endows those in the most privileged positions with the power and resources to attach connotations of excellence to the cultural practices they prefer (Costa and Murphy, 2015; Reeves, 2015). For example, legitimising classical over popular music or haute cuisine over fast food. Thus, according to Bourdieu, individuals occupying the same position within society share the same cultural tastes and practices (Bennett et al., 2008), first, because they are complicit in indicating their position and distinguishing themselves, and second, because they have been socialised to share a distinct habitus.

### Post-Distinction: Adaptations of Bourdieusian Theory and Methodology

Many scholars have explored the extent to which Bourdieu’s model of the cultural structure of 1960s French society can be applied to contemporary societies, in particular the USA (Lamont, 1992) and Britain (Bennett et al., 2008). Primarily, these analyses question the external validity of Bourdieu’s sample and methodology, but further whether political and social changes have restructured the social meaning of culture. For example, in Britain, educational reforms and political movements, such as Thatcherism and New Labour, have caused fundamental changes to class structure, with implications for the symbolism of culture (Bennett et al., 2008). There have therefore been many new appropriations of Bourdieusian theory (Julien, 2015; Kamphuis et al., 2015; Savage et al., 2013) that have garnered both support and criticism (Atkinson, 2012) for both Bourdieu *and* the applications of his work, generating continued debate and evolutions in discourse and approach.

For example, some repudiators of Bourdieu have criticised his anti-agentic assertions that tastes and practices are reflexive responses to socialisation; instead emphasising the agency individuals have over their interactions with culture (Costa et al., 2019). Others have argued that in current digital and globalised western societies, where access to cultural knowledge is uncoupled from educational systems, tastes are more evenly spread across societal factions (Bennett et al., 2008; Ostrower, 1998). For example, some have considered the role of the internet and broadcasting in disrupting the social meaning of culture, especially for younger people (Roose, 2015). Further, in the era of social media with hugely popular and influential online platforms composed of consumer-generated content (Julien, 2015) and algorithm-based advertising, status behaviours in younger people have never been more complex or de-institutionalised. However, Bourdieu controversially took little interest in other factors (such as age or gender) that may influence engagement, instead seeing cultural capital as the singular characterising *and characterised* feature of a status group (Bennett et al., 2008). By contrast, most contemporary explorations have discarded the conceptualisation of status groups as homogenous entities, and have explored the intersecting influences of age, gender, ethnicity and different indicators of social position, such as education, occupation and parental social position.

Methodologically, Bourdieu used MCA, popular at the time, to identify factors that influence the spatial distance between cultural taste indicators. For example, in *Distinction* we are shown a polarisation between ‘popular’ and ‘legitimate’ cultural practices (Bourdieu, 1981). However, this dichotomised view has been criticised for generating a falsely polarised perception of culture (Mohr, 2013). Instead, techniques such as LCA maintain the relational lens of an MCA but generate groupings that allow for composite contributions from each item. Therefore, the present analysis uses Bourdieusian theory to assess broad indicators of position in social space then latent class methodology to describe correlates of cultural engagement.

## Methodology

### Operationalising Cultural Engagement as a Behavioural Expression of Capital

The present analysis was conducted using data from *Understanding Society*, or the *UK Household Longitudinal Survey* (UKHLS), a household panel survey that began in 2009. Data are collected continuously from approximately 100,000 individuals from 40,000 households in the UK. Items from the survey’s Leisure, Culture and Sport module were used to conceptualise cultural practices as *behavioural expressions* of cultural tastes. This module was adapted from another survey, *Taking Part*, which was commissioned by the English government’s Department for Digital, Culture, Media and Sport in 2005 to assess cultural participation in England. Respondents were asked to indicate which of a series of cultural items (see Tables 1–3) they had engaged in over the previous 12 months. Both cultural consumption activities, such as gallery attendance, and production, such as painting, are included so that they may be compared. Although the barriers to engagement may differ between these two forms of cultural behaviours (Reeves, 2015), both may either require (Veenstra, 2007a) or generate capital (Stevens et al., 2008). Engagement may also better reflect the resources available to an individual, and the importance they attach to their tastes (Ostrower, 1998). Further, engagement can demonstrate an individual’s keenness to invest, or display, their resources (Yaish and Katz-Gerro, 2012). The cultural activities and covariates included in the present analysis have been guided by both Bourdieusian analyses (Bennett et al., 2008) and other LCAs of cultural engagement (Bunting et al., 2008; Mak et al., 2020; Reeves, 2015; Savage et al., 2013). However, to compare a breadth of status-related cultural activities in contemporary society, the analysis has not been limited to activities that occur within cultural institutions. Cultural indicators such as street arts, carnival attendance and snooker have also been included, representing a shift towards the ‘cultural democracy’ movement (Hadley and Belfiore, 2018), where creators and consumers are empowered to define what constitutes arts and culture. Therefore, patterns of engagement are assessed across a diverse range of activities, while excluding activities such as religious engagement that do not have clear connotations of distinction.

**Table 1.** Sports factors.

Sports factors	Individual items within factor	% of sample engaging over previous 12 months	
		At least one	More than one
Team	Hockey Basketball Volleyball Netball Baseball Athletics Gymnastics Cricket Rugby Racket sports	15.38	4.66
Outdoor	Walking Trekking Skiing Cycling Water sports Jogging	50.32	22.01
Traditional	Snooker Darts Football Golf	27.85	11.73
Country	Shooting Motor sports Fishing Archery	10.48	2.05
Leisure	Ice skating Bowling Swimming Horse riding	43.05	14.44
Fitness	Martial arts Boxing Fitness Yoga	31.79	6.90

**Table 2.** Arts factors.

Arts factors	Individual items within factor	% in sample engaging over previous 12 months	
		At least one	More than one
Institutional	Opera Classical music Ballet Plays Book clubs and events Exhibits Contemporary dance attendance Street arts	50.47	27.25
Creative	Painting/drawing Computer arts Photography Creative writing Textiles	37.86	15.79
Contemporary	Rock/pop/jazz concerts Circus attendance Circus performance Video arts event attendance	29.35	5.14
Choreographic	Carnival attendance Culturally specific festival attendance Culturally specific festival performance African/South Asian/Chinese dance participation Other dance participation	17.11	6.04
Performance	Writing music Playing musical instruments Singing Rehearsing or performing in plays Opera performance Musical theatre performance	14.91	4.39

**Table 3.** Heritage factors.

Heritage factors	Individual items within factor	% in sample engaging over previous 12 months	
		At least one	More than one
Reference	Public library Archive or records office use	36.99	2.99
Historic	Visited historic building Visited city or town Visited monument Visited place of worship Visited park Visited archaeological site Visited industrial site Visited museum or gallery Visited sports heritage site	66.24	40.78

### Data and Analytical Technique

Data from *Understanding Society* adult participants (aged 16–103) were accessed through the UK Data Service. Depending on availability through the waves, items from waves two (2011–2012) including socio-economic and economic capital indicators, and three (2012–2013) including cultural engagement items, social capital and demographics, were merged and treated as one wave. In the first, preliminary stage, Tetrachoric Factor Analyses (TFAs) were used to group cultural items in STATA (StataCorp, 2019). A TFA is a form of factor analysis for binary data that groups variables based on statistical covariation (Muthén and Hofacker, 1988). Then, the first two steps of a ‘three-step’ LCA, as favoured in the literature (Asparouhov and Muthén, 2014), were used: following the preliminary TFAs, the data were analysed in the advanced statistical software Mplus (Muthén and Muthén, 2011) by first specifying the model (LCA step one) then in the final stage, multinomial logistic regressions between estimated probability of class membership and correlates of engagement were conducted (LCA step two). This three-step approach reduces the influence of correlated factors on the model (Asparouhov and Muthén, 2014) and also uses estimated probability of class membership, instead of assigning individuals to classes, and so reduces the risk of misclassification error (Collier and Leite, 2017). Observations were dropped if data were missing across any of the cultural engagement items (*n* = 2,778) so a complete case was used (*n* = 41,397).

### Preliminary Step: Tetrachoric Factor Analyses

Separate TFAs were conducted for sports, arts then heritage activities. Positive semidefinite correlation matrices with oblique (for correlated data items) rotations were used and principal components were specified to ensure a solution was met given the particularly high sample size. The number of factors was unspecified and a factor loading cut-off of 0.3 was used, although all items loaded above this value, and none were excluded. Scree plots were used to confirm the number of factors (see online Appendix) although several modifications, outlined below, were trialled and incorporated if they made marked improvement to the LCA model fit and interpretability.

The sports TFA returned a seven-factor solution. The Kaiser-Meyer-Olkin (KMO) measure of sampling adequacy for the correlation was 0.92, indicating a very good fit. However, one of the factors contained two extremely unpopular sports (boules and croquet) and was excluded from the LCA. The arts TFA returned a six-factor solution, with a KMO of 0.90. Although one of these factors contained just one activity (dance participation) and factor loadings (see online Appendix) were used to move this item into its closest fitting factor, choreographic arts. The heritage TFA returned a two-factor solution, with a KMO of 0.94.

### Step One: LCA Model Optimisation

The 13 factors were turned into scalar variables indicating engagement in zero, one or more than one of the activities in each factor and exported to MPlus, for optimisation. First, the number of class sizes was increased from five to 15 and a six-class model was favoured for having the lowest number of classes, log-likelihood, Akaike Information Criterion, Bayesian Information Criterion, sample size adjusted Bayesian Information Criterion, Lo-Mendell-Rubin likelihood ratio test value (LMR-A) and bootstrapped likelihood ratio test (BLRT) while maintaining a high class entropy and significant LMR-A and BLRT p-values. Second, these fit statistics were used to see which factor modifications improved model fit and variation between classes. During this stage, particularly popular and non-discriminating activities were tested for exclusion. Removal of two items; reading for pleasure from the institutional arts factor and cinema attendance from the contemporary arts factor, made considerable improvements. Therefore, the final factors can be seen in Tables 1 to 3. For fit statistics, see the online Appendix.

### Step Two: Correlates of Class Membership

Cross-sectional multinomial logistic regressions between cultural engagement classes (as an estimated probability of membership) and indicators of economic and social capital, education, social class, parental education, parental social class and demographic factors were conducted. The model was fitted using maximum likelihood estimations with robust standard errors and Monte Carlo algorithm integrations.

### Operationalisation of Correlates of Cultural Engagement

Economic capital was operationalised using four variables: quintiles of total, net household income; four categories of estimated monthly savings (none or terciles); four categories of estimated house value (zero for non-homeowners then terciles); and a binary indicator of private or social housing (either local authority or housing association).

Social capital was operationalised using indicators at both individual and community levels. Eight variables covered objective and subjective measures of engagement at an individual level: marital/partnership status (including cohabitation); continuous number of close friends; four categories of interaction with family (zero or terciles of summed frequency of interaction with mother, father or children); continuous, summed, positive social support score (extent of feeling able to rely on, open up to or feel understood by friends, family and partner); and reverse scored social strain (extent of feeling let down, criticised or annoyed by friends, family and partner). Then, at a community level through civic organisational activities (engaging in zero, one or more than one of: political parties, trade unions, environmental groups, parent or school associations, tenants or residents groups, religious or church organisations, voluntary services groups, pensioner groups, scouts or guides organisations, professional organisations, other community groups, social or working men’s clubs, sports clubs, women’s institute or townswomen’s guilds, women’s or feminist groups). In addition, a derived variable indicating neighbourhood social cohesion using items from the Sampson Project on Human Development in Chicago Neighbourhoods (Sampson et al., 1997), such as ‘this is a close-knit neighbourhood’, is provided in the *Understanding Society* dataset and was used continuously.

Then, to address Bourdieusian notions of social group exclusivity and the role of mutuality of resource in determining social inclusion (Bourdieu, 1986; Costa et al., 2019), a score indicating social group homogeneity was generated from data on how similar the respondent reported themselves to be to their friends (proportion with similar income, education, occupational status, locality, ethnicity, age and friends that are also family members). For each, four item Likert scales were summed across each domain to generate a continuous score, where 28 indicates all friends are the same across each indicator.

Although Bourdieu conceptualised education as a form of cultural capital, in this analysis, education was considered an indicator of social position and was measured using three categories (higher educational qualifications, GCSE or equivalent and above, below GCSE) alongside social class (three-category National Statistics Socio-Economic Classification). In order to examine associations between engagement and an individual’s social origins, three categories of highest parental educational level and four categories of highest parental social class were additionally included.

Finally, age (six categories); binary gender and ethnicity (small group sizes necessitated use of white or non-white binarisation) were included alongside a series of additional potential barriers to engagement: current economic activity (self-employed, employed part time or full time (including maternity leave), unemployed, retired, full-time student, long-term sick, other (family care, unpaid family business, other)); caring for children under the age of 16; binary satisfaction with amount of leisure time; urbanicity; presence of a long-standing disability; being limited in moderate activities by health (binarised); and the openness to experience personality trait (continuous score).

## Results

In the final model, six latent classes of cultural engagement were identified and are shown in Table 4. Odds ratios showing the association between each latent class and the fully adjusted correlated variables are presented in Table 5. For each correlate, the lowest or least advantaged category was used as a reference, and the odds ratios are comparisons between the likelihood of membership of each class and the *Disengaged* class.

**Table 4.** Latent class model of cultural engagement.^a^

Name:		Disengaged			Low-Engagement			Recreational			Institutional & Historic			Sports			Omnivore		
Proportions based on estimated posterior probabilities:		***25.86***			***17.89***			***22.33***			***21.69***			***7.5***			***4.73***		
In each category:		0	1	1+	0	1	1+	0	1	1+	0	1	1+	0	1	1+	0	1	1+
Sports	Team	99	1	0	99	1	0	82	15	4	86	13	1	35	34	31	41	31	28
	Outdoor	91	9	0	59	37	4	47	35	18	20	42	38	7	22	70	6	14	80
	Traditional	91	7	2	94	5	0	55	27	18	78	17	5	14	26	60	35	31	34
	Country	97	3	0	97	3	0	87	11	2	91	8	1	64	27	10	68	21	11
	Leisure	92	8	1	79	19	2	40	44	16	40	43	17	12	36	53	10	35	55
	Fitness	94	6	0	86	13	1	61	34	6	54	34	13	32	53	16	23	47	30
Arts	Institutional	93	7	0	35	40	25	68	29	3	5	22	74	39	37	24	1	7	92
	Creative	87	12	2	56	31	13	71	20	9	41	29	30	55	24	21	17	21	63
	Performance	98	2	0	90	8	2	90	8	2	75	17	8	75	18	7	45	26	30
	Contemporary	97	4	0	84	16	0	73	26	1	48	33	9	46	46	8	13	41	46
	Choreographic	93	6	1	82	16	2	80	18	2	57	31	12	69	26	5	22	35	43
Heritage	Reference	82	18	0	58	39	4	71	29	1	44	50	7	63	36	2	34	55	11
	Historic	80	17	2	16	38	47	38	40	22	2	13	86	16	29	55	2	7	91

a Engagement is indicated as estimated proportion of class members engaging in none, one or more than one of the activities in the given factor, with red indicating a high % and blue a low %.

**Table 5.** Fully adjusted model of correlates of cultural engagement class membership. (Odds ratios for latent class membership use *Disengaged* as reference class.).

Name	Low-Engagement	Recreational	Institutional & Historic	Sports & Historic	Omnivore
Estimated size (%)	17.89	22.33	21.69	7.50	4.73
Economic capital					
Income (lowest quintile as reference)					
2nd quintile	1.09	1.20	1.27*	1.13	0.98
3rd quintile	1.13	1.32*	1.44*	1.26	0.87
4th quintile	1.28*	1.60**	1.80**	1.98*	1.70*
5th quintile	1.05	1.34*	1.60*	1.79*	1.47
Savings (none as reference)					
Low	1.42**	1.30*	1.92**	1.66*	2.16**
Medium	1.60**	1.58**	2.44**	2.36**	2.33**
High	2.10**	2.02*	3.42**	3.81**	3.64**
House value (renters as reference)					
Low	0.88	1.00	0.83*	1.15	0.49**
Medium	1.48*	1.87**	2.08**	2.38**	1.38*
High	1.65**	1.97**	3.17**	4.60**	2.59**
Private housing	1.96**	1.44*	2.75**	1.50*	3.18**
Social capital					
Partnered	1.05	0.58**	0.60**	0.39**	0.34**
Friendship (cont.)	1.02	1.04	1.04*	1.07**	1.07**
Family (no contact as reference)					
Low	1.27*	1.45**	1.88**	1.67*	1.88*
Medium	1.25*	1.27*	1.78**	1.93**	1.63*
High	1.29*	1.90**	1.90**	2.75**	2.33**
Positive support (cont.)	1.01*	1.03**	1.03**	1.03**	1.04**
Social strain (cont.)	1.02*	1.01	1.02*	1.02*	1.03*
Organisation (none as reference)					
1	1.74**	2.31**	2.95**	5.32**	3.46**
1+	3.56**	4.94**	12.75**	18.63**	28.32**
Neighbourhood (cont.)	0.97*	1.00	0.98	1.01	1.01
Homogeneity (cont.)	0.99	1.00	0.96**	0.99	0.94**
Education					
Low (reference)					
Medium	2.05**	1.65**	2.93**	2.42**	2.04**
High	3.11**	2.02**	7.40**	2.85**	5.18**
Social class					
Routine (reference)					
Intermediate	1.91**	1.83**	2.64**	2.23**	2.13*
Managerial/Prof.	2.10**	1.94**	4.28**	3.57**	3.78**
Highest parental educational level					
Low (reference)					
Medium	1.35*	1.40**	1.80**	1.47*	1.77*
High	1.59**	1.50**	2.79**	1.83**	3.05**
Highest parental social class					
Neither employed (reference)					
Routine	1.11	1.10	1.13	0.79*	0.67*
Intermediate	1.15	1.04	1.33*	0.77*	0.97
Managerial/Prof.	1.38*	1.17	1.97**	1.12	1.41*
Demographic					
Female gender (male as reference)	1.86**	0.44**	1.61**	0.11**	0.57**
Age (16–29 as reference)					
30 to 39	5.20	0.64**	1.58*	0.37**	0.54**
40 to 49	6.30	0.39**	1.69*	0.15**	0.38**
50 to 59	10.02*	0.23**	1.47*	0.04**	0.12**
60 to 69	14.89*	0.14**	1.56*	0.00**	0.06**
70 / max	11.43*	0.02**	0.46**	0.00#	0.00**
Ethnicity (non-white as reference)	2.84**	3.38**	8.31**	10.25**	8.79**
Current economic activity					
Unemployed (reference)					
In paid employment (inc. mat leave)	1.11	1.20	0.88	1.22	1.33
Self-employed	0.84	0.91	0.69*	1.82	1.70
Retired	1.71*	0.92	1.66*	4.88	1.22
Home care and other	1.31	0.70*	1.02	0.44**	1.37
Student	7.12	3.39**	7.03**	9.27**	14.13**
Long-term sick or disabled	0.96	0.37**	0.40**	0.06**	0.17**
Additional correlates					
Parent	0.92	1.27*	0.942	1.33*	0.70*
Leisure time satisfaction	1.04	1.08	1.14*	1.22*	1.26*
Urban	1.17*	1.12	1.24*	0.99	1.40*
Disability	1.05	0.88*	0.89	0.79*	0.97
Limited by health	0.73**	0.63**	0.42*	0.47**	0.46**
Open personality	1.35**	1.12**	1.85**	1.32**	2.63**

# extreme value approached, value fixed at 0.00, *p* = 0.00 to stabilise model. Bold text represents odds ratios that are statically significant, either at the p < 0.001 or p < 0.05 threshold.

** *p* < 0.001, *p < 0.05.

### Latent Class Model of Cultural Engagement

Table 4 is a heatmap indicating the proportion of the members of each of the six classes engaging in zero, one or more than one of the activities in each factor, where red represents a high proportion. With the exception of low engagement in heritage and creative activities, members of the first class, *Disengaged*, were noticeably disengaged in every activity, even the popular factors (outdoor sports, institutional arts and historic heritage). This was the largest latent class, with an estimated proportion of 25.9% of respondents, although is much smaller than the disengaged classes found in other LCAs (Bunting et al., 2008; Mak et al., 2020; Reeves, 2015). The second class, *Low-Engagement*, was composed of respondents who reported overall low levels of engagement yet moderate engagement in the popular activities: outdoor sports, institutional and creative arts, and heritage activities. This was the third smallest class, constituting 17.9% of the sample. The third class, *Recreational*, contained 22.3% of the sample and had some moderate engagement in recreational activities like outdoor, traditional, leisure and fitness sports, as well as historic heritage and institutional arts. While the other classes generally followed a consistent pattern of increasing engagement across the activities, 21.7% of the sample were in the *Institutional & Historic* class and their activity was characterised by very high engagement in *only* institutional and historic activities. The fifth class, *Sports & Historic*, was characterised by generally high engagement in the sports activities and low engagement in the arts (except institutional and creative). This was a smaller class, representing 7.5% of the sample. Finally, the smallest class, with an estimated percentage membership of 4.7%, was the *Omnivore* class. This class had the highest proportion of engagement in more than one of each factor, with particularly high engagement in outdoor and leisure sports, institutional and creative arts, and historic heritage activities. Most notably, this class had high engagement in performance, contemporary and choreographic arts – which were unpopular in all other classes.

### Correlates of Cultural Engagement Latent Class Membership

Table 5 shows the fully adjusted odds ratios for each correlate of engagement, when compared with the *Disengaged* class. In this mutually adjusted multivariate model, economic and social capital, adult indicators of social position and demographic factors were all independently associated with cultural engagement. Increasing capital was generally associated with increased engagement, although a less consistent pattern was seen for income and neighbourhood cohesion. Increased age, ethnic minority identity and having a long-term illness or disability were all generally associated with lower levels of engagement.

Members of the *Low-Engagement* class had middling levels of economic and social capital and were more likely to be female, older, more educated and were 71% more likely to be retired. This class also emerged as the least socio-economically advantaged after the *Disengaged* class. Members of the *Recreational* class were more likely to be male, younger and responsible for children under the age of 16. Members of the *Institutional & Historic* class had consistently highly significant odds of not being in the least advantaged category for each covariate, and were more likely to live in urban areas. The *Sports & Historic* class represented another wealthy and advantaged class. In particular, they had the highest overall levels of economic capital and were the most likely to be young, white (10.3 times increased odds) and male (89% increased odds). However, members of the *Sports & Historic* class were not notably more educated than the *Institutional & Historic* or *Omnivore* classes, or even the *Low-Engagement* class. There was also no relationship between *Sports & Historic* membership and having a parent in managerial or professional employment. Finally, the *Omnivore* class was less consistently associated with capital or position and more strongly correlated with being under 30 years old, white and having an open personality (2.6 times increased odds for each unit increase on the scale). Members were also the most likely to have heterogeneous social groups with 6% reduced odds of membership for each unit increase in homogeneity, and most likely to be full-time students (14.1 times increased odds).

## Discussion

This multistep LCA identified six distinct classes of cultural engagement. Some of the findings support those seen in previous research; however, this analysis offers a more comprehensive understanding of cultural engagement patterns and their association with indicators of capital, social position and other aspects of identity. Generally, previous work has shown engagement classes to vary quantitatively by the extent of cultural engagement (Bunting et al., 2008; Devine and Dowds, 2013; Mak et al., 2020; Reeves, 2015). While also seen here, the inclusion of a range of arts, cultural, sports and heritage items allowed this analysis to look broadly and identify some *qualitative* differences. For example, the *Sports & Historic* class was characterised by greater engagement in sports, *as well as* historic heritage activities and low arts engagement. This patterning would not have been identifiable in the previous LCA models, which were limited to exploration of each type of activity separately. Therefore, this model offers new insight on how the wider scope of activities, such as sports or contemporary arts engagement, may fit alongside more traditionally aristocratic cultural activities to reflect cultural capital and status behaviours in distinct ways.

Upon inclusion of the correlates, each class became more distinctly defined, supporting Bourdieusian theory that capital, position and social identity constructs influence patterns of engagement. In line with other LCAs, a clear pattern between advantage and overall cultural engagement emerged, demonstrating that social and economic disadvantage may act as barriers to engagement (Bennett et al., 2008; Bunting et al., 2008; Devine and Dowds, 2013; Hanquinet et al., 2014; Mak et al., 2020; Reeves, 2015; Roose, 2015; Veenstra, 2007b). However, the three most advantaged and engaged classes, *Institutional & Historic, Sports & Historic* and *Omnivore*, each had distinct positional and cultural characteristics. For example, members of the *Institutional & Historic* class were engaging frequently in a narrow range of activities, each connotated with sufficient leisure time, ticketing or travel costs and cultural aristocracy. Perhaps wealth, cohort and life-stage effects could explain their narrow cultural tastes, and this class may represent the most traditional, Bourdieusian cultural distinction behaviours. By contrast, the *Omnivores*, who were socially advantaged but younger, appeared to indicate their status by developing a preference for a broad range of activities (Peterson and Kern, 1996). The *Omnivores* were also the most likely to be students, suggesting immersion into academic institutions is a key determinant of high levels of diverse cultural engagement. Perhaps, as proposed in De Vries and Reeves’s (2021) development of the Omnivore hypothesis, universities generate a culturally egalitarian habitus, characterised by a rejection of class-based, aristocratic structures of cultural legitimacy. It is of note that these young, yet advantaged, students will likely go on to have high-grade, powerful occupations, where omnivorous cultural engagement will be a key characteristic of their elite habitus. Thus, this form of engagement will be canonised as both legitimate and indicative of high social status.

While each of the more engaged classes shared distinction-related behaviours, such as institutional arts and historic heritage participation in common, members of the *Sports & Historic* class had distinct correlates of engagement, which may explain their preference, or capability, for sporting activities. For example, they were more likely to live in rural areas, were the youngest and were the least likely to have disabilities, long-term conditions or limitations in moderate activities. However, we should also consider how sports engagement may display status. Sports are often conceptualised as ‘emergent’ forms of cultural engagement (Prieur and Savage, 2013; Veenstra, 2007b), and are therefore not hypothesised to be associated with social advantage, or distinction behaviours (Bourdieu, 1981). By contrast, this analysis found engagement in sports was most strongly correlated with economic advantage, and also with being male and white. Further, despite being wealthy, the members of this class were less likely to have high parental social position. Therefore, we might consider that high levels of familial capital could guide individuals to invest their economic capital into culturally elite activities, while these socially mobile, young, wealthy men pursue sports and tourism. Identifying this characteristic group offers an opportunity to question the symbolic or social value of different activities for different social agents. As discussed in Roose’s (2015) analysis of emerging cultural capital and young people, this could highlight how an individual’s experience of the cultural field is *micro*, and dependent on their habitus and the legitimacy their particular social group attaches to given activities. In this case, these men may not gain position in their field by indicating their creativity but could profit through sporting skill or travelling experience. Again, it is worth considering the occupational or societal positions these individuals will likely attain, and the role shared cultural tastes may play in determining who belongs in (and benefits from) their social and professional networks.

In support of the literature on cultural engagement and personality (Batey and Furnham, 2006; Fancourt and Tymoszuk, 2019), openness to experience was strongly correlated with membership of the more engaged classes. However, a personality trait such as openness may be considered an ongoing social reflection, or disposition, or to Bourdieu, part of an individual’s *habitus* (Costa, 2013). Openness is likely to result from parental interactions, socialisation and education that foster curiosity and confidence (McCrae and Costa, 1997), which themselves are likely to reflect social position. Further, conceptualising openness as a feature of the habitus invites us to consider the role of agency in cultural engagement. What may appear to be a choice or natural predisposition to engage based on personality traits, may in fact be reflexive responses to socialisation and privilege. The habitus that is shared by members of a social strata is likely to consciously or subconsciously determine, or certainly influence, distinction behaviours, yet appear as choice and preference.

Strengths of the analysis include the use of a very large and representative dataset, and a comprehensive yet theoretically informed approach, with particular regards to social covariates. Very few cultural activities were excluded, and the analysis included a range of ‘culturally democratic’ (Hadley and Belfiore, 2018) activities, that were scaled empirically. This approach further means the model could be applied to other datasets to identify context-specific variations in engagement patterns and predictors, independent of researcher bias. Future research could further explore the apparently strong influence of age in determining cultural engagement class by using longitudinal data to test whether engagement varies by life-course stage or if cohort effects or social mobility are responsible. However, the findings of this analysis contrast the existing longitudinal literature, which has demonstrated younger cohorts are less likely to be omnivores (Van Steen et al., 2015). In addition, much work is needed in understanding the role of ethnicity in likelihood to engage; particularly in discerning whether the effects seen reflect psychological (or practical) barriers. It is likely for example, that the measures of cultural engagement are still too specific to mainstream, white, British culture and may not capture what cultural engagement means across different socio-cultural and ethnic groups. This is likely exacerbated by the use of the cultural engagement module adapted from the *Taking Part* survey, which despite its diversity, is still rooted within institutional, aristocratic definitions of culture. Further, despite being minimised, some subjectivity will have been introduced by using three tetrachoric factor analyses, and the dropping of observations that were missing across all cultural engagement variables may have been non-random. Also, the data may have self-report bias, and the questionnaire design meant frequency could not be applied to each form of cultural engagement. Therefore, the analysis is limited to binary frequency over the 12-month period.

In conclusion, this LCA looked across a broad range of cultural activities and established six distinct classes of engagement. In support of capital theory and the Omnivore hypothesis, breadth of engagement increased with advantage. However, different forms of capital or position were found to correlate with particular engagement patterns. We may therefore consider the symbolic power of different cultural activities, such as sports, within particular social fields. Overall, it is a comprehensive model in a high-quality dataset that offers greater insight than previously available models and highlights the plausibility of cultural engagement as a driving factor in the manifestation of social stratification.

## Supplemental Material

sj-docx-1-soc-10.1177_00380385221130163 – Supplemental material for A Bourdieusian Latent Class Analysis of Cultural, Arts, Heritage and Sports Activities in the UK Representative Understanding Society Dataset Click here for additional data file. Supplemental material, sj-docx-1-soc-10.1177_00380385221130163 for A Bourdieusian Latent Class Analysis of Cultural, Arts, Heritage and Sports Activities in the UK Representative Understanding Society Dataset by Emma S Walker, Daisy Fancourt, Feifei Bu and Anne McMunn in Sociology
