# Supplementary material for: A Bourdieusian Latent Class Analysis of Cultural, Arts, Heritage and Sports Activities in the UK Representative Understanding Society Dataset
Source: Sociology. 2022 Nov 13;57(4):843–64. doi: 10.1177/00380385221130163 (PMC10623679; doi:10.1177/00380385221130163)
Supplement: sj-docx-1-soc-10.1177_00380385221130163 – Supplemental material for A Bourdieusian Latent Class Analysis of Cultural, Arts, Heritage and Sports Activities in the UK Representative Understanding Society Dataset [file sj-docx-1-soc-10.1177_00380385221130163.docx]

**Online Appendix:** A Bourdieusian Latent Class Analysis of cultural, arts, heritage and sports activities in the UK representative Understanding Society dataset

#### Tetrachoric factor analysis scree plots


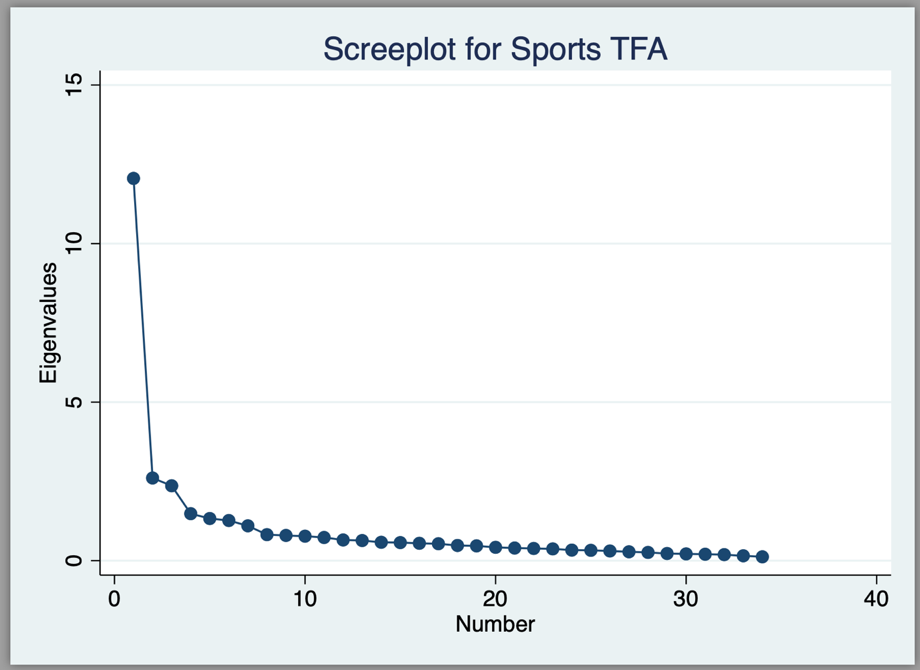


Figure 1: Scree plot for sports tetrachoric factor analysis


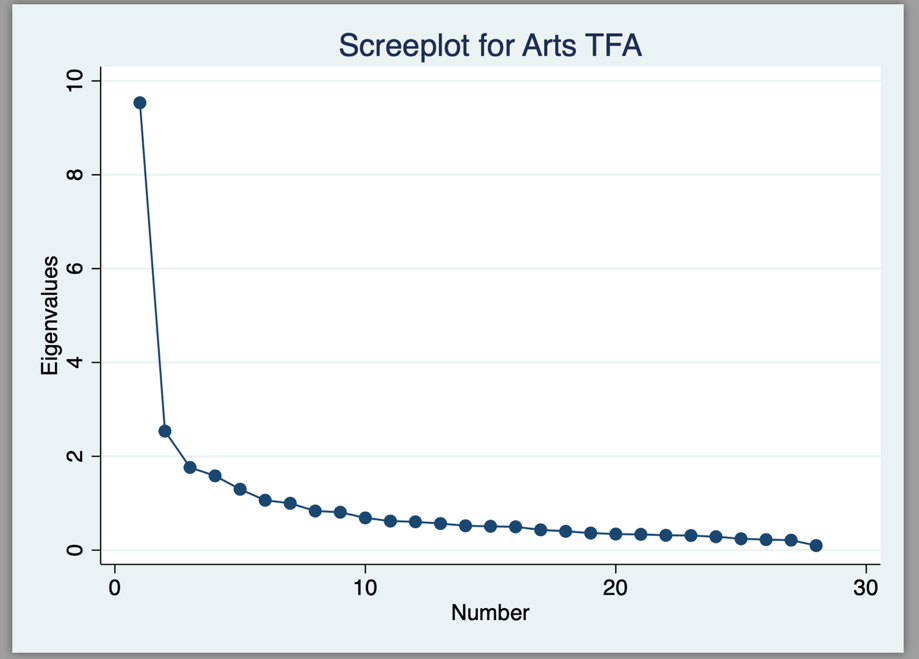


Figure 2: Scree plot for arts tetrachoric factor analysis


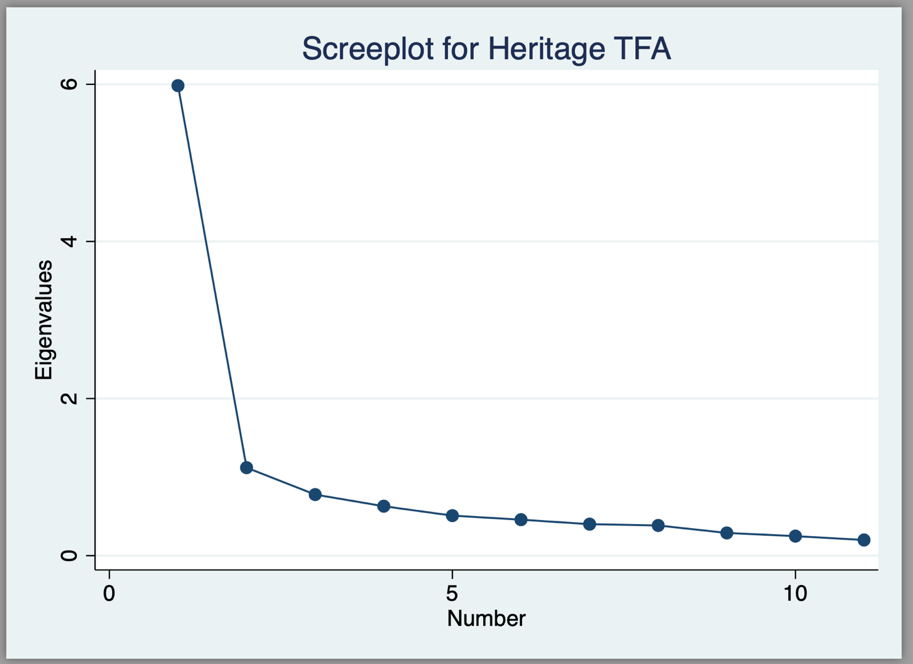


Figure 3: Scree plot for Heritage tetrachoric factor analysis

#### Full TFA outputs

Table 1: Sports TFA full factor loadings

| Item | Sports factors | | | | | | | Uniqueness |
| --- | --- | --- | --- | --- | --- | --- | --- | --- |
|  | Team | Outdoor | Traditional | Country | Recreational | Fitness | Lawn |  |
| Fitness | 0.3052 | 0.3519 | 0.1657 | -0.1159 | 0.2753 | **0.5586** | -0.0843 | *0.3472* |
| Gymnastics | **0.6193** | 0.0461 | -0.0910 | 0.0921 | 0.0160 | 0.2958 | 0.0174 | *0.5096* |
| Swimming | 0.1595 | 0.4483 | 0.2374 | 0.0024 | **0.5482** | 0.1368 | -0.0431 | *0.3962* |
| Cycling | 0.2196 | **0.5879** | 0.2635 | 0.2415 | 0.1659 | 0.1710 | -0.1157 | *0.4082* |
| Football | 0.5192 | 0.0455 | **0.6753** | 0.1729 | -0.0530 | 0.1668 | -0.1010 | *0.2016* |
| Rugby | **0.6038** | 0.1702 | 0.3432 | 0.2977 | -0.0421 | 0.1997 | -0.0383 | *0.3569* |
| Athletics | **0.7519** | 0.2292 | 0.2006 | 0.1901 | 0.0743 | 0.2593 | -0.0696 | *0.2282* |
| Jogging | **0.4423** | 0.4053 | 0.2903 | 0.0098 | 0.1944 | 0.3756 | -0.1535 | *0.3532* |
| Trekking | 0.2404 | **0.7136** | 0.0077 | 0.2512 | 0.0330 | 0.1227 | 0.0978 | *0.3441* |
| Golf | 0.1879 | 0.2991 | **0.6285** | 0.1509 | -0.0115 | -0.0681 | 0.0943 | *0.4438* |
| Boxing | 0.3895 | - 0.0195 | 0.2761 | 0.2636 | 0.0831 | **0.6864** | -0.0781 | *0.2181* |
| Martial Arts | 0.2150 | 0.0672 | 0.0549 | 0.2245 | 0.0388 | **0.7419** | 0.1275 | *0.3277* |
| Water Sports | 0.3250 | **0.5823** | 0.1020 | 0.4000 | 0.1983 | 0.0869 | 0.0020 | *0.3380* |
| Horse riding | 0.1178 | 0.2318 | -0.2228 | 0.3174 | **0.5230** | 0.1349 | -0.0605 | *0.4866* |
| Basketball | **0.7979** | 0.0051 | 0.2996 | 0.1165 | 0.1153 | 0.1550 | 0.0435 | *0.2207* |
| Netball | **0.7673** | 0.0981 | -0.0738 | - 0.0739 | 0.3810 | 0.0467 | 0.1521 | *0.2203* |
| Volleyball | **0.7973** | 0.1687 | 0.2222 | 0.1092 | 0.1786 | 0.1020 | 0.0603 | *0.2286* |
| Cricket | **0.6075** | 0.1231 | 0.4816 | 0.1125 | -0.0406 | 0.0482 | 0.0590 | *0.3638* |
| Hockey | **0.8014** | 0.1844 | 0.1446 | 0.0780 | 0.2173 | 0.0438 | 0.0823 | *0.2408* |
| Baseball | **0.7537** | 0.1508 | 0.2139 | 0.1145 | 0.2531 | 0.0581 | 0.1490 | *0.2607* |
| Racquet | **0.4928** | 0.4357 | 0.3518 | -0.0262 | 0.1338 | 0.0749 | 0.2050 | *0.3773* |
| Ice-skating | 0.3525 | 0.1000 | 0.1181 | 0.1305 | **0.6738** | 0.0903 | -0.0159 | *0.3724* |
| Snow Sports | 0.3215 | **0.6357** | 0.0704 | 0.1891 | 0.1256 | -0.0096 | -0.1109 | *0.4237* |
| Motor Sports | 0.1269 | 0.1240 | 0.2921 | **0.5802** | 0.1203 | 0.2091 | -0.1383 | *0.4693* |
| Fishing | 0.0371 | 0.1707 | 0.3095 | **0.5787** | -0.0117 | -0.0504 | 0.0495 | *0.5336* |
| Snooker | 0.1925 | 0.0400 | **0.7509** | 0.2357 | 0.2338 | 0.1553 | 0.1556 | *0.2389* |
| Darts | 0.1620 | 0.0204 | **0.7104** | 0.2296 | 0.1673 | 0.0803 | 0.1496 | *0.3592* |
| Bowling | 0.2114 | 0.0920 | 0.4191 | 0.0514 | **0.6397** | 0.0924 | 0.1769 | *0.3195* |
| Walking | -0.1035 | **0.7299** | 0.0253 | 0.0032 | 0.1216 | 0.0513 | 0.2534 | *0.3743* |
| Shooting | 0.1393 | 0.1845 | 0.1559 | **0.7253** | 0.0975 | 0.0680 | 0.2328 | *0.3279* |
| Yoga | 0.0172 | 0.3975 | -0.2162 | -0.3215 | 0.3101 | **0.5351** | 0.0758 | *0.3034* |
| Croquet | 0.0016 | 0.4636 | 0.1134 | 0.0377 | -0.0380 | 0.0537 | **0.5535** | *0.4601* |
| Archery | 0.2813 | 0.1170 | 0.0377 | 0.5362 | 0.2593 | 0.1584 | **0.4173** | *0.3518* |
| Bowls | 0.1419 | -0.0244 | 0.1457 | 0.1104 | 0.0161 | -0.0227 | **0.7463** | *0.3880* |

Table 2: Arts TFA full factor loadings – for dance both dance and choreographic factors are emboldened as the dance item was moved to the choreographic factor to improve LCA fit.

| Item | Arts factors | | | | | | *Uniqueness* |
| --- | --- | --- | --- | --- | --- | --- | --- |
|  | Institutional | Creative | Performance | Choreographic | Contemporary | Dance |  |
| Dance | 0.1328 | 0.1348 | 0.2137 | **0.2521** | 0.1504 | **0.6137** | *0.4557* |
| Singing | 0.2926 | 0.1161 | **0.7615** | 0.1457 | -0.0036 | 0.2129 | *0.2545* |
| Playing music | 0.1201 | 0.2443 | **0.7831** | 0.0390 | 0.1850 | - 0.0373 | 0.2755 |
| Writing music | 0.0140 | 0.2569 | **0.8797** | 0.1100 | 0.1703 | - 0.1103 | 0.1068 |
| Performing arts | 0.2638 | 0.1433 | **0.6900** | 0.1785 | 0.0607 | 0.3222 | 0.2943 |
| Carnival - performance | 0.0606 | 0.2115 | 0.3790 | **0.6697** | 0.0554 | 0.2560 | 0.2908 |
| Circus - performance | -0.0300 | 0.3314 | **0.3306** | 0.2227 | 0.3634 | 0.2641 | 0.5286 |
| Film | 0.3056 | 0.0905 | 0.1101 | 0.1707 | **0.7153** | 0.0665 | 0.3411 |
| Art exhibit | **0.6441** | 0.3999 | 0.0709 | 0.2288 | 0.3045 | - 0.1275 | 0.2588 |
| Video art | 0.3044 | **0.4100** | 0.2015 | 0.3605 | 0.4146 | - 0.2455 | 0.3365 |
| Book Event | **0.5940** | 0.4111 | 0.1259 | 0.2538 | 0.1454 | - 0.1442 | 0.3560 |
| Street Art | **0.4400** | 0.3676 | 0.0542 | 0.3869 | 0.4050 | - 0.1229 | 0.3394 |
| Attending Carnivals | 0.0706 | 0.1158 | 0.0368 | **0.7831** | 0.2312 | 0.0363 | 0.3122 |
| Paint | 0.0425 | **0.7435** | 0.1475 | 0.0863 | 0.1239 | 0.2613 | 0.3326 |
| Photography | 0.1520 | **0.6814** | 0.2141 | 0.1531 | 0.1010 | - 0.0298 | 0.4322 |
| Computer Art | -0.0164 | **0.7189** | 0.2271 | 0.1812 | 0.1168 | -0.0128 | 0.3846 |
| Craft | 0.2801 | **0.5598** | - 0.0529 | 0.0015 | - 0.0401 | 0.4309 | 0.4180 |
| Reading | **0.5380** | 0.3559 | 0.0872 | - 0.0955 | 0.2761 | 0.1105 | 0.4788 |
| Writing | 0.2144 | **0.5631** | 0.3689 | 0.1386 | 0.0402 | 0.0137 | 0.4798 |
| Book club | **0.6444** | 0.3005 | -0.0257 | 0.0924 | -0.0108 | 0.0321 | 0.4841 |
| Play | **0.6477** | -0.0003 | 0.1419 | 0.0763 | 0.4315 | 0.2362 | 0.3125 |
| Opera | **0.8088** | -0.0445 | 0.1725 | 0.0674 | 0.0017 | 0.0118 | 0.3094 |
| Classical music | **0.7633** | 0.0521 | 0.2878 | 0.1056 | 0.0185 | -0.0447 | 0.3183 |
| Rock concert | 0.1757 | 0.1078 | 0.2859 | 0.1390 | **0.6623** | -0.1676 | 0.3897 |
| Contemporary dance | 0.4526 | 0.0788 | 0.1698 | **0.4389** | 0.2012 | 0.2719 | 0.4531 |
| Ballet | **0.7296** | -0.0450 | 0.0499 | 0.1316 | 0.0809 | 0.2526 | 0.3755 |
| African etc. dance | 0.2457 | 0.1589 | 0.0982 | **0.6784** | 0.1060 | 0.0766 | 0.4273 |

Table 3: Heritage TFA full factor loadings

| Item | Heritage Factors | | Uniqueness |
| --- | --- | --- | --- |
|  | Historic | Reference |  |
| Library | 0.0693 | **0.8632** | *0.2501* |
| Archive | 0.2496 | **0.7376** | *0.3936* |
| Museum | **0.6641** | 0.4383 | *0.3668* |
| Town | **0.8552** | 0.1800 | *0.2361* |
| Building | **0.8705** | 0.2134 | *0.1966* |
| Park | **0.7910** | 0.1995 | *0.3344* |
| Industrial | **0.7248** | 0.1923 | *0.4377* |
| Worship | **0.7936** | 0.1949 | *0.3323* |
| Monument | **0.8348** | 0.1611 | *0.2771* |
| Archaeological sites | **0.7893** | 0.1447 | *0.3561* |
| Sports heritage | **0.5310** | 0.0528 | *0.7152* |

#### Model fitting

Table 4: Initial fit statistics for the original factors.

| No. of classes | Log-likelihood | AIC | BIC | ssaBIC | Entropy | LMR-A | LMR-A *p*-value | BLRT loglikelihood | BLRT *p*-value |
| --- | --- | --- | --- | --- | --- | --- | --- | --- | --- |
| 5 | -409,228.958 | 818,755.915 | 820,041.929 | 819,568.406 | 0.700 | 3,331.580 | 0.0000 | -410,899.971 | 0.0000** |
| **6** | **-408,319.902** | **816,997.804** | **818,542.746** | **817,973.883** | **0.695** | **1,812.427** | **0.0000** | **-409,228.957** | **0.0000**** |
| 7 | -407,697.988 | 815,813.975 | 817,617.847 | 816,953.644 | 0.665 | 1,239.940 | 0.0000 | -408,319.902 | 0.0000** |
| 8 | -407,163.675 | 814,805.351 | 816,868.151 | 816,108.608 | 0.648 | 1,065.284 | 0.0000 | -407,697.988 | 0.0000** |
| 9 | -406,780.517 | 814,099.034 | 816,420.763 | 815,565.879 | 0.639 | 763.922 | 0.2472 | -407,163.675 | 0.0000** |
| 10 | -406,410.179 | 813,418.357 | 815,999.015 | 815,048.792 | 0.656 | 738.361 | 0.0175 | -406,780.517 | 0.0000** |
| 11 | -406,159.349 | 812,976.698 | 815,816.285 | 814,770.722 | 0.651 | 500.091 | 0.1919 | -406,410.179 | 0.0000** |
| 12 | -405,957.725 | 812,633.451 | 815,731.967 | 814,591.063 | 0.645 | 401.987 | 0.4873 | -406,159.349 | 0.0000** |
| 13 | -405,794.744 | 812,367.489 | 815,724.934 | 814,488.690 | 0.629 | 324.943 | 0.1985 | -405,957.725 | 0.0000* |
| 14 | -405,658.800 | 812,155.601 | 815,771.975 | 814,440.390 | 0.625 | 271.038 | 0.6355 | -405,794.744 | 0.0000* |
| 15 | -405,520.507 | 811,939.014 | 815,814.316 | 814,387.392 | 0.630 | 260.122 | 0.3623 | -405,650.976 | 0.0000** |

***5 bootstrap draws did not converge, p-value may not be trustworthy*

**100 bootstrap draws did not converge, p-value may not be trustworthy*

Table 5:Fit statistics for the latent class model iterations.

| EXCLUDING (or modified): | No. of classes | Log-likelihood | AIC | BIC | ssaBIC | Entropy | LMR-A | LMR-A *p*-value | BLRT loglikelihood | BLRT *p*-value |
| --- | --- | --- | --- | --- | --- | --- | --- | --- | --- | --- |
| None | 7 | -407697.988 | 815813.975 | 817617.847 | 816953.644 | 0.665 | 1239.94 | 0 | -408319.902 | 0.0000* |
| Lawn sports | 7 | -400471.314 | 801332.628 | 803015.666 | 802395.955 | 0.663 | 1156.705 | 0 | -401051.609 | 0.0000* |
| Reading | 7 | 406291.916 | 813001.832 | 814805.703 | 814141.500 | 0.678 | 1078.668 | 0 | -406832.941 | 0.0000* |
| Walking | 7 | -400483.099 | 801384.198 | 803188.070 | 802523.867 | 0.666 | 1158.73 | 0 | -401064.281 | 0.0000* |
| Cinema | 7 | -397077.879 | 794573.758 | 796377.629 | 795713.426 | 0.664 | 1153.75 | 0 | -397656.563 | 0.0000* |
| Town | 7 | 408043.899 | 816505.799 | 818309.67 | 817645.467 | 0.666 | 1314.569 | 0 | -408703.245 | 0.0000* |
| Reading, cinema, lawn | 5 | -389841.107 | 779960.214 | 781159.918 | 780718.175 | 0.693 | 2820.882 | 0 | -391256.286 | 0.0000* |
| Reading, cinema, lawn | 6 | -389003.658 | 778341.316 | 779782.687 | 779251.96 | 0.688 | 1669.29 | 0 | -389841.107 | 0.0000* |
| Reading, cinema, lawn | 7 | -388514.373 | 777418.746 | 779101.784 | 778482.073 | 0.665 | 975.293 | 0.0698 | -389003.658 | 0.0000* |
| Reading, cinema, walking | 5 | -389930.47 | 780158.939 | 781444.952 | 780971.43 | 0.708 | 2716.689 | 0 | -391293.073 | 0.0000* |
| Reading, cinema, walking | 6 | -389026.55 | 778411.093 | 779956.035 | 779387.172 | 0.697 | 1802.195 | 0 | -389930.469 | 0.0000* |
| Reading, cinema, walking | 7 | -388549.217 | 777516.434 | 779320.305 | 778656.102 | 0.669 | 951.672 | 0.5164 | -389026.545 | 0.0000* |
| Reading, cinema, lawn, dance | 5 | -376115.591 | 752499.181 | 753655.730 | 753229.877 | 0.696 | 2786.413 | 0 | -377513.837 | 0.0000* |
| Reading, cinema, lawn, dance | 6 | -375350.847 | 751023.695 | 752413.280 | 751901.621 | 0.688 | 1524.176 | 0 | -376115.591 | 0.0000* |
| Reading, cinema, lawn, dance | 7 | -374863.263 | 750102.525 | 751725.146 | 751127.681 | 0.664 | 971.652 | 0 | -375350.846 | 0.0000* |
| Reading, cinema, lawn  and dance in choreo | 5 | -381903.678 | 764075.357 | 765231.906 | 764806.053 | 0.694 | 2804.164 | 0 | -383310.645 | 0.0000* |
| **Reading, cinema, lawn and dance in choreo *(*final mode)** | **6** | **-381110.658** | **762543.315** | **763932.900** | **763421.241** | **0.688** | **1580.535** | **0** | **-381903.678** | **0.0000*** |
| Reading, cinema, lawn  and dance in choreo. | 7 | -380627.783 | 761631.566 | 763254.187 | 762656.722 | 0.663 | 962.396 | 0.0014 | -381110.658 | 0.0000* |

**5 bootstrap draws did not converge, p-value may not be trustworthy*
